# Correction: Burnout among anesthesia personnel in Thailand: A national survey of workload, personality traits, and resilience

**DOI:** 10.1371/journal.pone.0354956

**Published:** 2026-07-28

**Authors:** Napasorn Sakulteera, Pongtong Puranitee, Atipong Pathanasethpong, Sirirat Rattana-arpa, Kasana Raksamani

No authors should have been attributed equal contribution to this work.

## References

[1] Sakulteera N, Puranitee P, Pathanasethpong A, Rattana-Arpa S, Raksamani K. Burnout among anesthesia personnel in Thailand: A national survey of workload, personality traits, and resilience. PLoS One. 2026;21(5):e0349336. doi: 10.1371/journal.pone.0349336 42133718 PMC13175325

