# Supplementary material for: Burnout among anesthesia personnel in Thailand: A national survey of workload, personality traits, and resilience
Source: PLoS One. 2026 May 14;21(5):e0349336. doi: 10.1371/journal.pone.0349336 (PMC13175325; doi:10.1371/journal.pone.0349336)
Supplement: Attachment — Submitted filename: RESPONSE TO EDITOR AND REVIEWERS.docx [file pone.0349336.s002.docx]

**RESPONSE TO EDITOR AND REVIEWERS**

Manuscript Title: **Burnout among anesthesia personnel in Thailand: a national survey of workload, personality traits, and resilience**
Manuscript ID: PONE-D-25-33433

We sincerely thank the Editor and Reviewers for their constructive comments and positive evaluation of our manuscript. We have carefully revised the manuscript in accordance with all journal and reviewer requirements. Below, we provide a detailed point-by-point response.

**Response to journal requirements**

1. PLOS ONE Formatting Requirements

Comment:

Please ensure that the manuscript meets PLOS ONE style requirements.

Response:

We have carefully revised the manuscript to ensure full compliance with PLOS ONE formatting guidelines. Specifically:

The title page and author affiliations were reformatted according to PLOS ONE templates.

Section headings were standardized.

Figures and tables were reformatted to match journal style.

File naming conventions were adjusted according to submission requirements.

The revised manuscript now adheres fully to the PLOS ONE formatting templates.

2. Funding information and acknowledgments

Comment:

Funding information should not appear in the Acknowledgments section. Please remove funding-related text and clarify the Funding Statement.

Response:

We thank the Editor for this clarification.

All funding-related text has been removed from the Acknowledgments section.

The Acknowledgments section now includes only expressions of gratitude for statistical consultation, research coordination support, and participant contribution.

The Funding Statement remains as follows:

This research project was supported by the Faculty of Medicine Siriraj Hospital, Mahidol University, under Grant Number (IO) R016731019. The award was received by K.R. (Kasana Raksamani). The funder had no role in the study design, data collection and analysis, decision to publish, or preparation of the manuscript.

We confirm that funding information appears exclusively in the Funding Statement section.

3. Data Availability Statement

Comment:

Please clarify restrictions on data sharing or upload anonymized data to a repository.

Response:

This study involves survey data from human participants (anesthesia personnel). Although the dataset has been de-identified, certain demographic variables (e.g., workplace context, professional role, and combination of personal characteristics) may potentially allow indirect identification within a relatively small professional community.

Data sharing restrictions were imposed by the Institutional Review Board of the Faculty of Medicine Siriraj Hospital, Mahidol University, in accordance with institutional ethical policy and participant consent conditions.

Data are available upon reasonable request to:

Institutional Review Board

Faculty of Medicine Siriraj Hospital, Mahidol University

Bangkok, Thailand

Email: sirb.ec4@gmail.com

The Data Availability Statement has been updated to:

Due to ethical restrictions related to participant confidentiality and institutional policy, the de-identified dataset is available upon reasonable request to the Institutional Review Board of the Faculty of Medicine Siriraj Hospital, Mahidol University.

4. Reviewer-Suggested Citations

Comment:

Review and evaluate recommended citations.

Response:

We carefully reviewed all suggested citations. Relevant publications have been incorporated where appropriate to strengthen theoretical grounding and contextualization. No citations were added that were not directly relevant to the study.

5. Reference List Review

Comment:

Ensure references are complete and correct; address any retracted articles.

Response:

The reference list has been carefully reviewed and updated. We confirm:

- All references are current and accurate.
- No retracted articles are cited.
- Minor formatting inconsistencies were corrected.
- Any additions or modifications are reflected in the revised manuscript.

**Response to reviewer comments**

Reviewer #1

We sincerely thank Reviewer #1 for the positive evaluation and constructive suggestions.

Comment 1: Clarification of classification and profile analysis

Reviewer comment:

The classification and technicalities of the profile analysis need clarification. There is limited theoretical justification for the profiles, and it is unclear how profiles were obtained (e.g., Latent Class Profile?).

Response:

We thank the reviewer for this important comment and agree that further clarification was needed regarding the burnout profile classification.

We would like to clarify that the burnout profiles in this study were not derived using latent class analysis (LCA), latent profile analysis (LPA), or cluster-based statistical modeling. Rather, we applied the theoretical burnout profile framework proposed by Maslach and Leiter, which categorizes individuals based on predefined cut-off values across the three MBI-HSS dimensions: emotional exhaustion (EE), depersonalization (DP), and personal accomplishment (PA).

Specifically:

- Participants were classified into the following profiles based on whether each dimension fell into high or low ranges according to established thresholds:
  - Engaged
  - Ineffective
  - Overextended
  - Disengaged
  - Burnout
- This approach is theory-driven and categorical, not data-driven.
- No latent modeling, model fit indices (e.g., AIC/BIC), or entropy-based classification procedures were performed.

To address the reviewer’s concern, we have revised the *Materials and Methods* section to explicitly describe:

1. The theoretical foundation of the Maslach burnout profile framework.
2. The operational criteria used for assigning participants to each profile.
3. The distinction between descriptive profile classification and the binary “high-risk burnout” variable used in regression analyses.

We have also clarified in the *Results* section that the burnout profile distribution was presented as a descriptive characterization of burnout patterns in the sample, while multivariable logistic regression was conducted using the predefined high-risk burnout definition (EE ≥27 and/or DP ≥10).

These revisions improve methodological transparency and clarify that the profile analysis was grounded in established theory rather than latent statistical modeling.

**Comment 2:** Logistic Regression – Marginal Errors and Classification Accuracy

Reviewer comment:
Include marginal errors and classification accuracy for better interpretation.

Response:

We thank the reviewer for this helpful suggestion.

To improve model interpretability and assess overall performance, we conducted receiver operating characteristic (ROC) analysis using the predicted probabilities from the multivariable logistic regression model. The model demonstrated good discriminatory ability, with an area under the curve (AUC) of 0.868 (95% CI: 0.84–0.89). These results have now been incorporated into the Results section.

Regarding marginal effects, we retained adjusted odds ratios as the primary effect estimates, as they represent the conventional and widely accepted reporting standard for logistic regression in epidemiological research. Given that the primary aim of our analysis was to identify independent predictors rather than to develop a predictive probability model, we believe the presentation of adjusted odds ratios provides appropriate and clinically interpretable measures of association.

Reviewer #2

We thank Reviewer #2 for the positive evaluation and thoughtful reflections.

**Comment:** Reliability Concerns (Time Constraints, Mentality/Ethnic Factors, Subjective Responses)

Response:

We appreciate this thoughtful observation and agree that contextual and subjective factors may influence responses in survey-based research. We have clarified in the Limitations section that time constraints, sociocultural influences, and the self-reported nature of the instruments may affect response reliability. We also emphasized that findings should be interpreted within the Thai healthcare context and warrant validation in other settings.

We sincerely thank the Editor and Reviewers for their constructive comments and positive evaluation. We believe the revisions have strengthened the methodological transparency, interpretability, and theoretical grounding of the manuscript. We respectfully submit the revised version for your consideration.
